# Supplementary material for: A new mouse model of ATR-X syndrome carrying a common patient mutation exhibits neurological and morphological defects
Source: Hum Mol Genet. 2023 May 12;32(15):2485–501. doi: 10.1093/hmg/ddad075 (PMC10360390; doi:10.1093/hmg/ddad075)
Supplement: Table_S1_corrected_ddad075 [file table_s1_corrected_ddad075.pdf]

Table S1: Primers

**Primer name**	**Sequence**
ATRX_RC gen F	GTTGTCTTTTGCCACAAACCCT
ATRX_RC gen R	CTCAAATACGCTGTTGCACG
qATRX gDNA F	GGTGGGAACTTGATTTGTTGTG
qATRX gDNA R	AGACAACTCCTTTCGACCAAG
qTFRC gDNA F	CTAAGTCTACAGTGGCTGTATTCC
qTFRC gDNA R	ATCATTGATTTCCCTCATGACAAA
qATRX F	AGCTTGTGGACAGCAGGTCAA
qATRX R	AAATTCTCAAATACGCTGTTGCACG
qGAPDH F	CGGCAAATTCAACGGCACAG
qGAPDH R	TCACAAACATGGGGGCATCG
qHba-a1/2 F	CGTGTGGATCCCGTCAACT
qHba-a1/2 R	TACTTGGAGGTCAGCACGGT
qHbb F	ACCTTTGCCAGCCTCAGTG
qHbb R	GAAATCCTTGCCCAGGTGGT
