# Supplementary material for: A new mouse model of ATR-X syndrome carrying a common patient mutation exhibits neurological and morphological defects
Source: Hum Mol Genet. 2023 May 12;32(15):2485–501. doi: 10.1093/hmg/ddad075 (PMC10360390; doi:10.1093/hmg/ddad075)
Supplement: Supplementary_figures_and_legends_corrected_ddad075 [file supplementary_figures_and_legends_corrected_ddad075.pdf]

Supplementary figures and legends

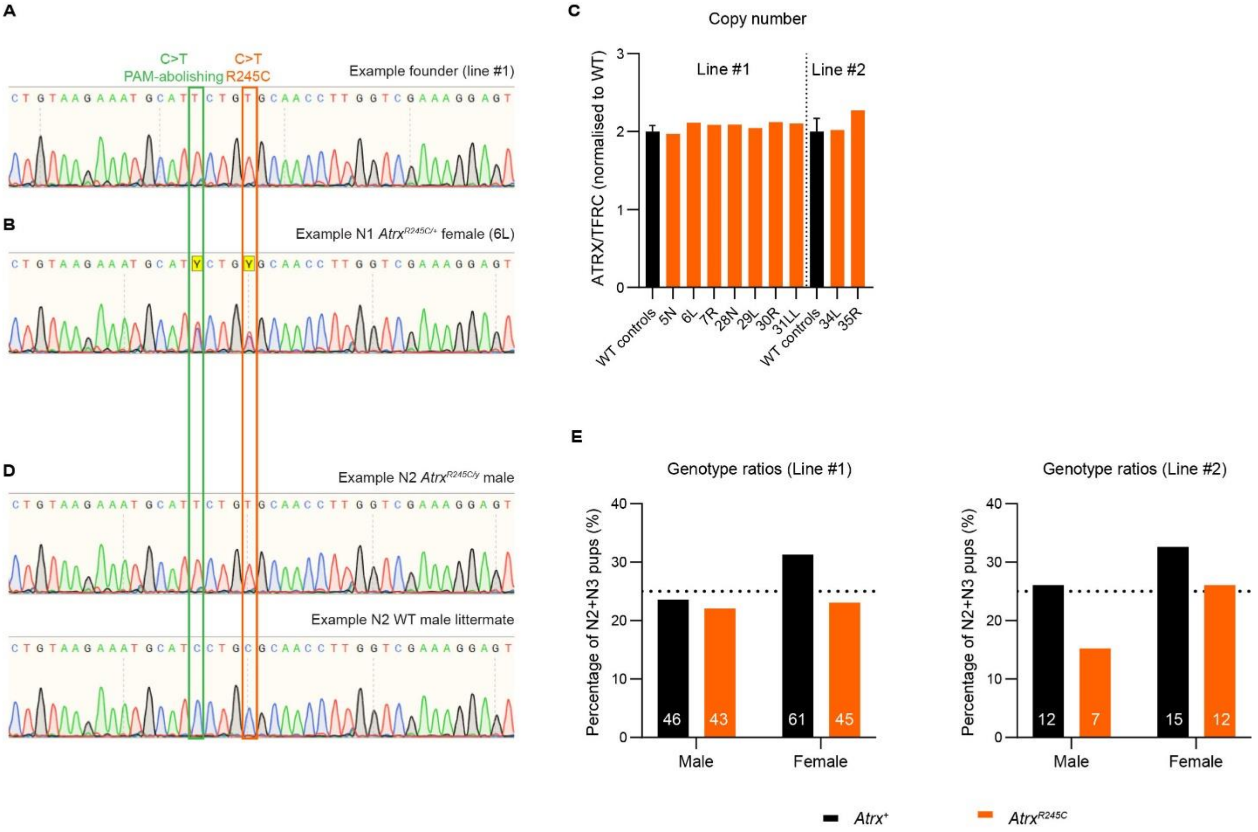

Figure S1. Production of *Atrx*$^{R245C/y}$ knock-in mice

A. Example Sanger sequencing read from the founder used to establish line #1 (gDNA from tail clip). **B**. Example Sanger sequencing read from an N1 *Atrx*$^{R245C/+}$ female, #6L. **C.** qPCR analysis of gDNA using primers within the donor molecule indicate no additional insertions in N1 *Atrx*$^{R245C/+}$ females in lines #1 and #2. **D.** Example Sanger sequencing reads from an N2 *Atrx*$^{R245C/y}$ male (upper) and WT littermate (lower). **E.** Genotype ratios (%) of N2 and N3 pups in lines #1 and #2 (n numbers on bars). Ratios are not significantly different from expected Mendelian ratios: line #1 P = 0.47; line #2 P = 0.76 (Fisher’s exact tests).

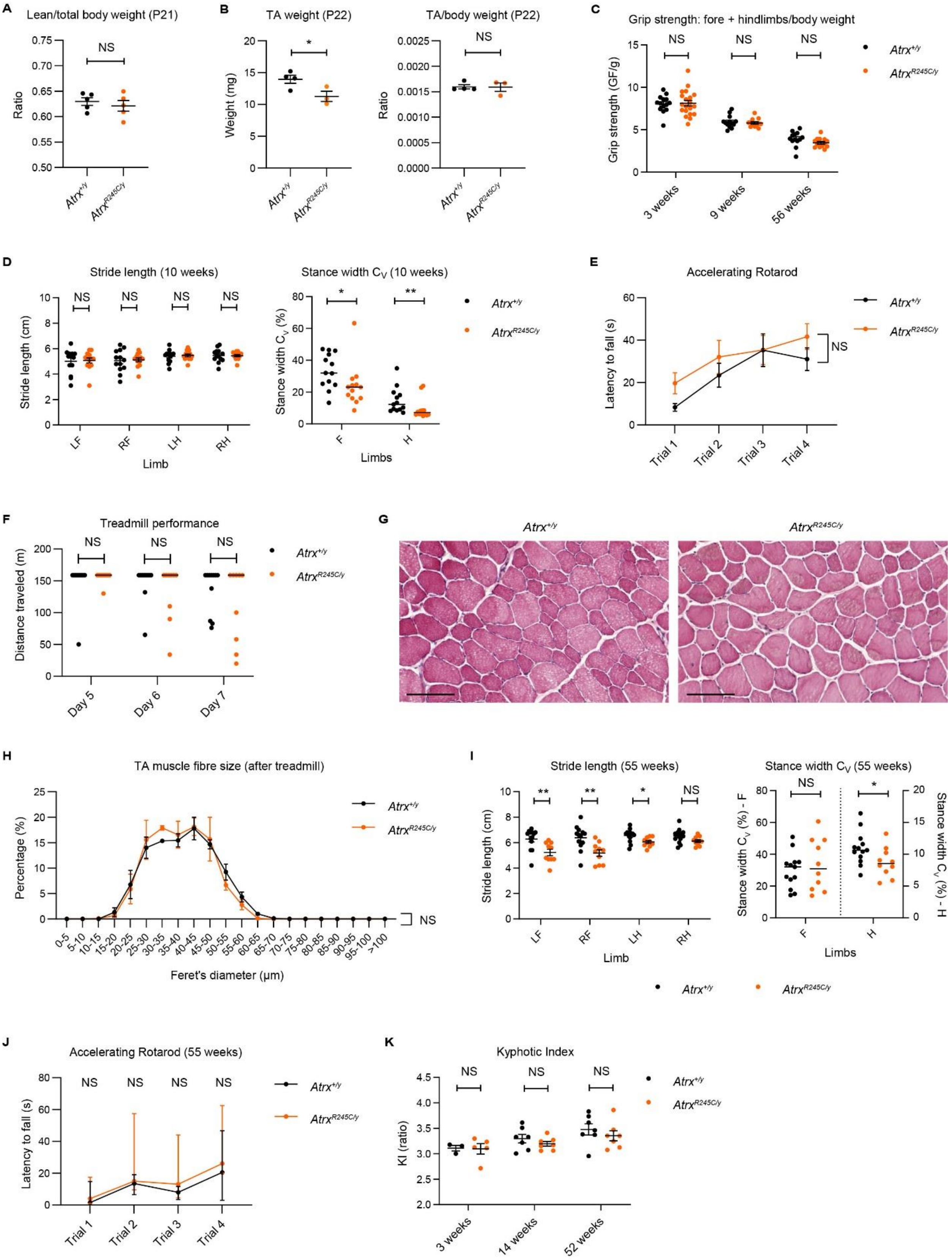

Figure S2. ***Atrx$^{R245C/y}$* mice do not display muscle defects** *(legend on next page)*

Figure S2. ***Atrx**$^{R245C/y}$* **mice do not display muscle defects**

A. DEXA analysis of lean/total body weight ratios at P21 (WT n = 5; *R245C* n = 5). Graph shows mean ± S.E.M. and genotypes were compared using a t-test: P > 0.05. **B.** Tibialis anterior (TA) muscle weights (right) and normalised to body weight (left) at P25 (WT n = 4; *R245C* n = 3). Graphs show mean ± S.E.M. and genotypes were compared using t-tests: TA weight * P = 0.046; TA/body weight P > 0.05. **C.** Forelimb and hindlimb grip strength (normalised to body weight) at 3 weeks (WT n = 14; *R245C* n = 20), 9 weeks (WT n = 13; *R245C* n = 12), and 52 weeks (WT n = 13; *R245C* n = 13). Graph shows mean ± S.E.M. and genotypes were compared using t-tests for each cohort: all P > 0.05. **D.** Gait assessed using motorised treadmill at 19 cm/s: stride length for each paw (left) and stance width coefficient of variation (C_V) for front and hind paws (right) at 10 weeks (WT n = 13; *R245C* n = 13). Stride length: graph shows mean ± S.E.M. and genotypes were compared using t-tests: NS P > 0.05. Stance width C_V: graph shows medians and genotypes were compared using KS tests: Fore (F) * P = 0.046; Hind (H) ** P = 0.004. **E.** Latency to fall from the accelerating rotarod in four trials over one day at 13 weeks (WT n = 13; *R245C* n = 13). Graph shows mean ± S.E.M. and genotypes were compared using repeated measures ANOVA: NS P > 0.05. Both genotypes show improvement over the course of the day, analysed by 1-way ANOVAs: WT ** P = 0.003; *R245C* ** P = 0.003. **F.** Performance on treadmill during trials (days 5-7) at 13 weeks (WT n = 11; *R245C* n = 9). Trials were ended early if animals showed signs of exhaustion. Graph shows median and genotypes were compared using Mann-Whitney tests: all P > 0.05. **G.** Representative images of WT (left) and RC (right) tibialis anterior (TA) muscle harvested after chronic exercise, stained with H&E. Scale bar: 100 µm. **H.** Histogram of Feret's diameter measured in TA muscle after chronic exercise (WT n = 3; *R245C* n = 3). Graph shows mean ± S.E.M. and genotypes were compared using repeated measures ANOVA: P > 0.05. **I.** Gait analysis (as **D**) of an independent cohort at 55 weeks (WT n = 13; *R245C* n = 10). Stride length: graph shows mean ± S.E.M. and genotypes were compared using t-tests: Left fore (LF) ** P = 0.005; Right fore (RF) ** P 0.004; Left hind (LH) * P = 0.015; Right hind (RH) P = 0.055. Stance width C_V: graph shows medians and genotypes were compared using t-tests: Fore (F) P > 0.05; Hind (H) * P = 0.045. Note: 3/13 mutants were unable to complete the task so were excluded.

J. Accelerating rotarod analysis (as **E**) of an independent cohort at 55 weeks (WT n = 8; *R245C* n = 9). Graph shows median ± interquartile range and genotypes were compared by KS tests: all P > 0.05. Both genotypes show improvement over the course of the day, analysed by Freidman tests: WT ** P = 0.009; R245C ** P = 0.001. **K**. Kyphotic indices measured from X-rays at 3 weeks (WT n = 3; *R245C* n = 5), and 14 and 52 weeks (WT n = 7; *R245C* n = 7). Graph shows mean ± S.E.M. and genotypes were compared using t-tests for each time point: all P > 0.05.

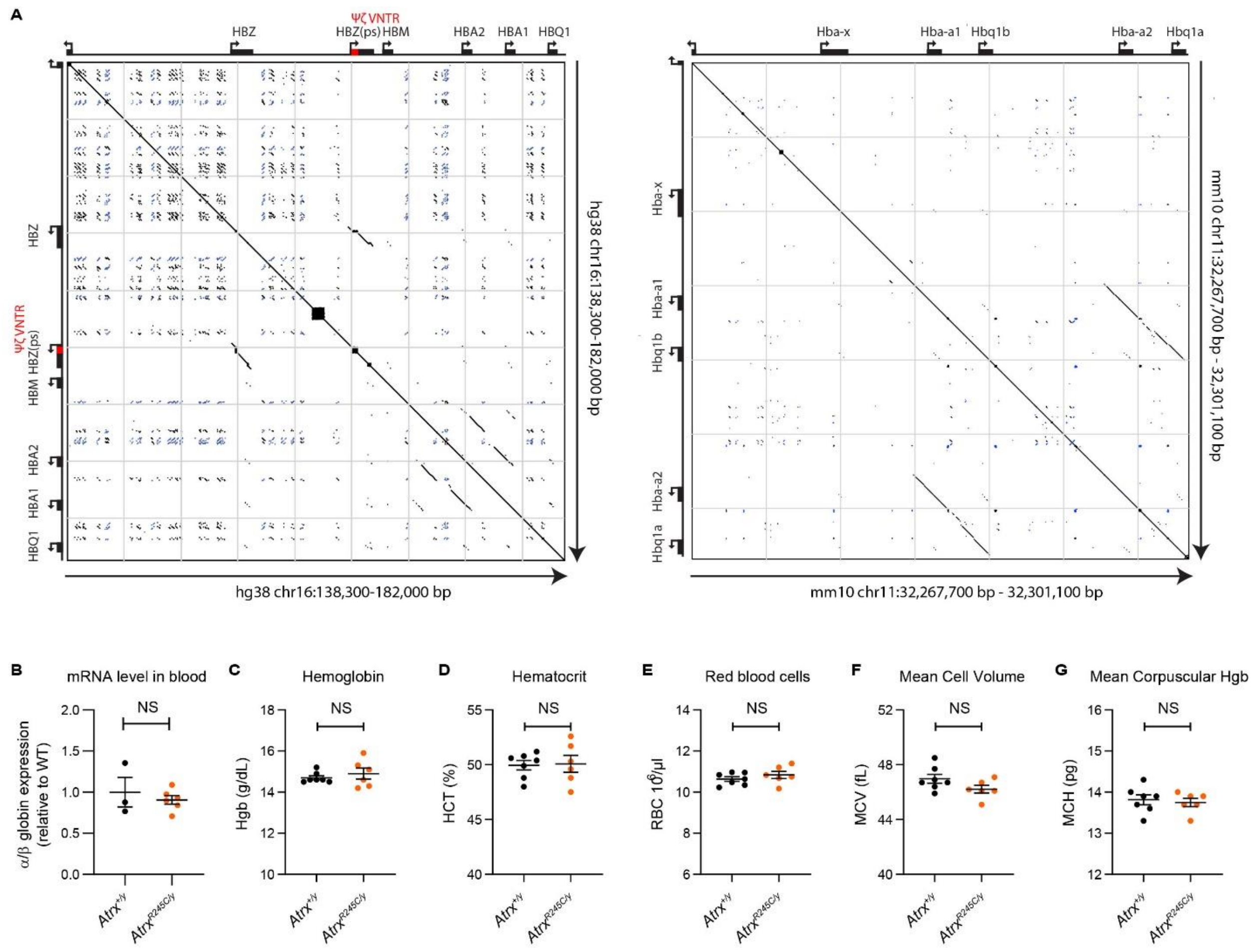

Figure S3: ***Atrx$^{R245C/y}$*** **mice do not develop alpha-thalassemia**

A. Analysis of self-similarity in the human (left) and mouse (right) alpha-globin loci. Dot plots showing self-alignment of a ~44 kb region from human chromosome 16 and a ~33 kb region from mouse chromosome 11. Both regions show the region from the *NPRL3* promoter to 3' of the distal paralogous alpha-globin gene. Diagonals represent regions of sequence identity (self-identity diagonal is shown centrally). Black diagonals show identity in the forward orientation and blue diagonals show identity in the reverse orientation. **B.** qPCR analysis of alpha/beta globin expression in blood from *Atrx$^{R245C/y}$* mice and WT controls at 18 weeks (WT n = 3; *R245C* n = 6). Graph shows mean ± S.E.M. and genotypes were compared using a t-test: NS P > 0.05. **C-G.** Complete blood count analysis at 14 weeks (WT n = 7; *R245C* n = 6). Graphs show mean ± S.E.M. and genotypes were compared using t-tests: all NS P > 0.05.

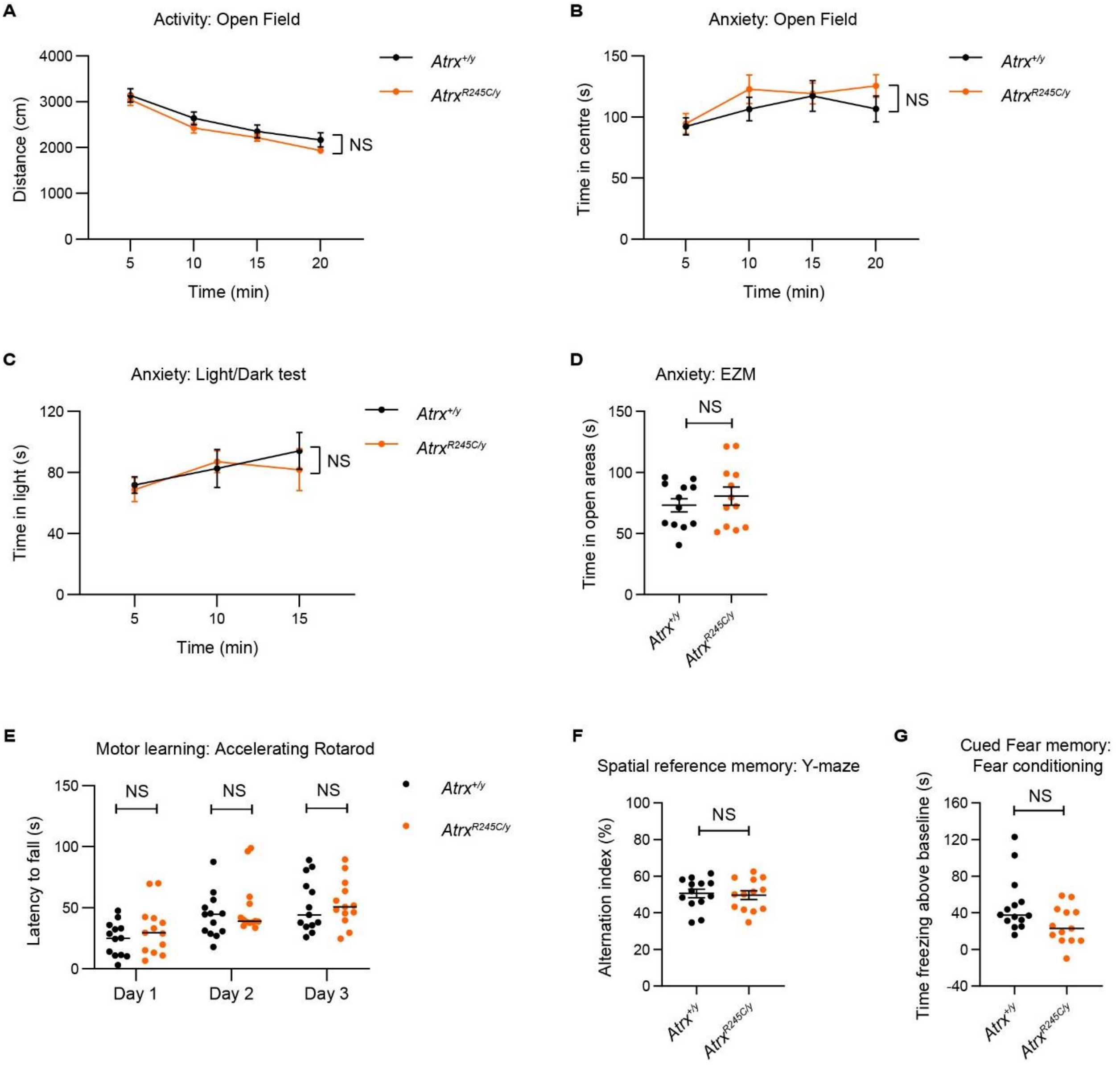

Figure S4. ***Atrx***$^{R245C/y}$ **mice display normal activity, anxiety, motor learning, spatial reference memory and cued fear memory**

A-B. Distance travelled (**A**) and time spent in the centre (**B**) in the Open Field test over 20 min (as 5 min bins) at 9 weeks of age (WT n = 12; *R245C* n = 13). Graphs show mean ± S.E.M. and genotypes were compared using repeated measures ANOVA: both P > 0.05. **C.** Time spent in the light half of the light/dark test over 15 min (as 5 min bins) at 10 weeks (WT n = 13; *R245C* n = 13). Graph shows mean ± S.E.M. and genotypes were compared using repeated measures ANOVA: P > 0.05. **D.** Time spent in open areas of the elevated zero maze (EZM) over 5 min at 9 weeks (WT n = 12; *R245C* n = 12). Graph shows mean ± S.E.M. and genotypes were compared using a t-test: P > 0.05. **E.**

Performance over three days on the accelerating rotarod at 13 weeks (continued from Figure S2E). Means of four daily trials are shown per animal and the line denotes group median (WT n = 13; *R245C* n = 13). Genotypes were compared on each day using KS tests: all NS P > 0.05. Both genotypes show learning over the three days of the experiment, analysed by Freidman tests: WT *** P = 0.0008; *R245C* * P = 0.037. **F.** Spatial reference memory was assessed over 8 min in the Y-maze test at 12 weeks (WT n = 13; *R245C* n = 13). Alternation index = number of alternations/max alternations * 100. Graph shows mean ± S.E.M. and genotypes were compared using a t-test: P > 0.05. **G.** Cued fear conditioning analysis at 13 weeks (WT n = 14; *R245C* n = 13). Time spent freezing after hearing a tone (minus baseline before tone) that accompanied the foot shock 24 h previously. Graph shows median and genotypes were compared using a KS test: P > 0.05.

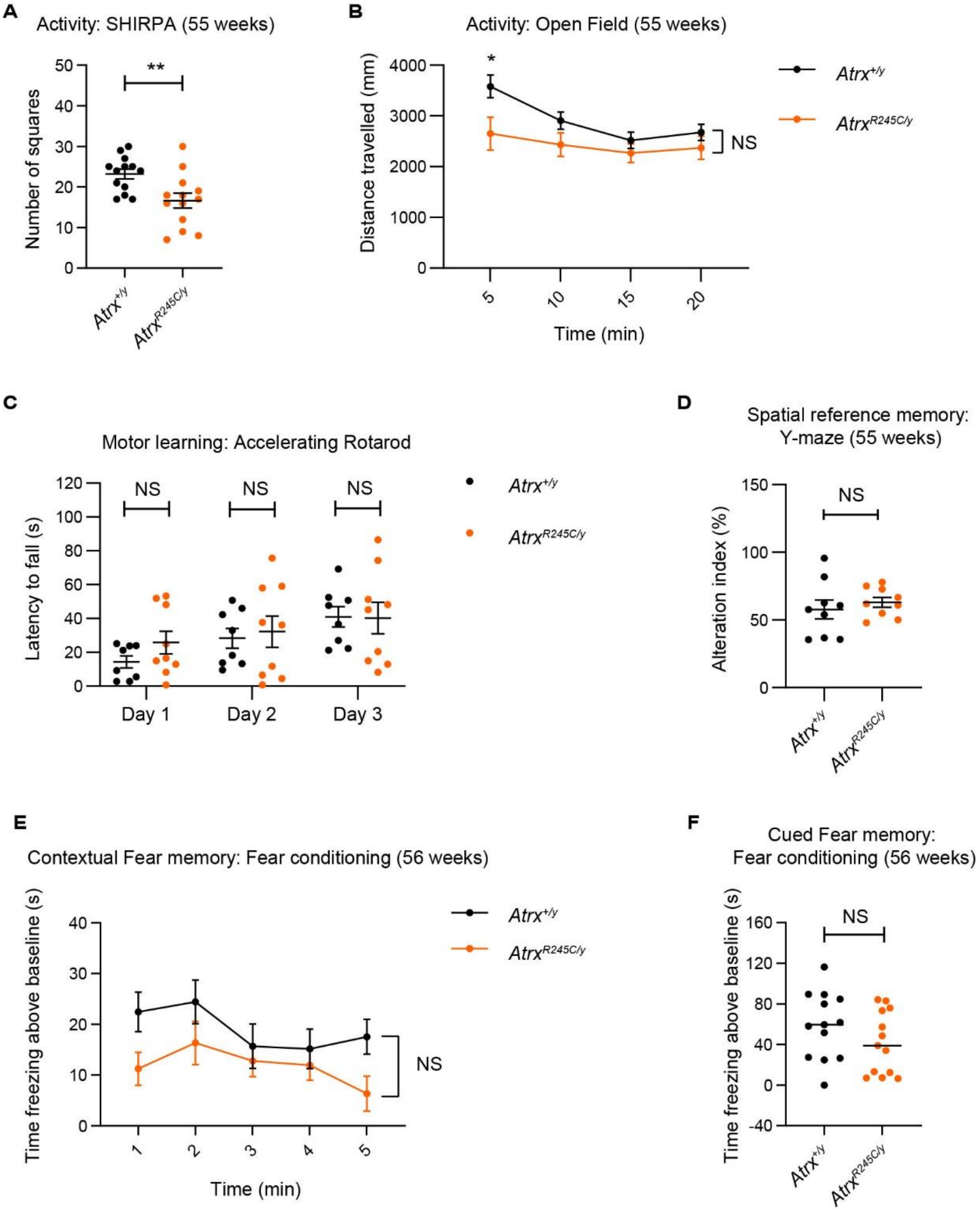

Figure S5. ***Atrx*$^{R245C/y}$ mice do not develop late onset memory defects**

A. Spontaneous activity assessed by number of squares entered in 30 s repeated at 55 weeks (WT n = 13; *R245C* n = 13). Graph shows mean ± S.E.M. and genotypes were compared using a t-test: ** P = 0.006. **B.** Distance travelled over 20 min (as 5 min bins) in the Open Field test at 55 weeks (WT n = 9; *R245C* n = 9). Graph shows mean ± S.E.M. and genotypes were compared using repeated measures ANOVA: P > 0.05. Individual time bins were compared by t-tests and mutants show hypoactivity in

bin 1: * P = 0.031. **C.** Performance over three days on the accelerating rotarod at 55 weeks (continued from Figure S2J). Means of four daily trials are shown per animal and the line denotes group mean ± S.E.M. (WT n = 8; *R245C* n = 9). Genotypes were compared on each day using t-tests: all NS P > 0.05. Only WT animals show learning over the three days of the experiment, analysed by 1-way ANOVA: WT ** P = 0.006; *R245C* P > 0.05. **D.** Spatial reference memory was assessed over 8 min in the Y-maze test at 55 weeks (WT n = 9; *R245C* n = 9). Alternation index = number of alternations/max alternations * 100. Graph shows mean ± S.E.M. and genotypes were compared using a t-test: P > 0.05. **E-F.** Fear conditioning analysis at 56 weeks (WT n = 13; *R245C* n = 13). **E.** Contextual: time spent freezing (minus baseline determined before shock) when returned to the same environment 24 h after receiving a foot shock. Graph shows mean ± S.E.M. and genotypes were compared using repeated measures ANOVA: P > 0.05. **F.** Cued: time spent freezing after hearing a tone (minus baseline before tone) that accompanied the foot shock 24 h previously. Graph shows median and genotypes were compared using a t-test: P > 0.05.

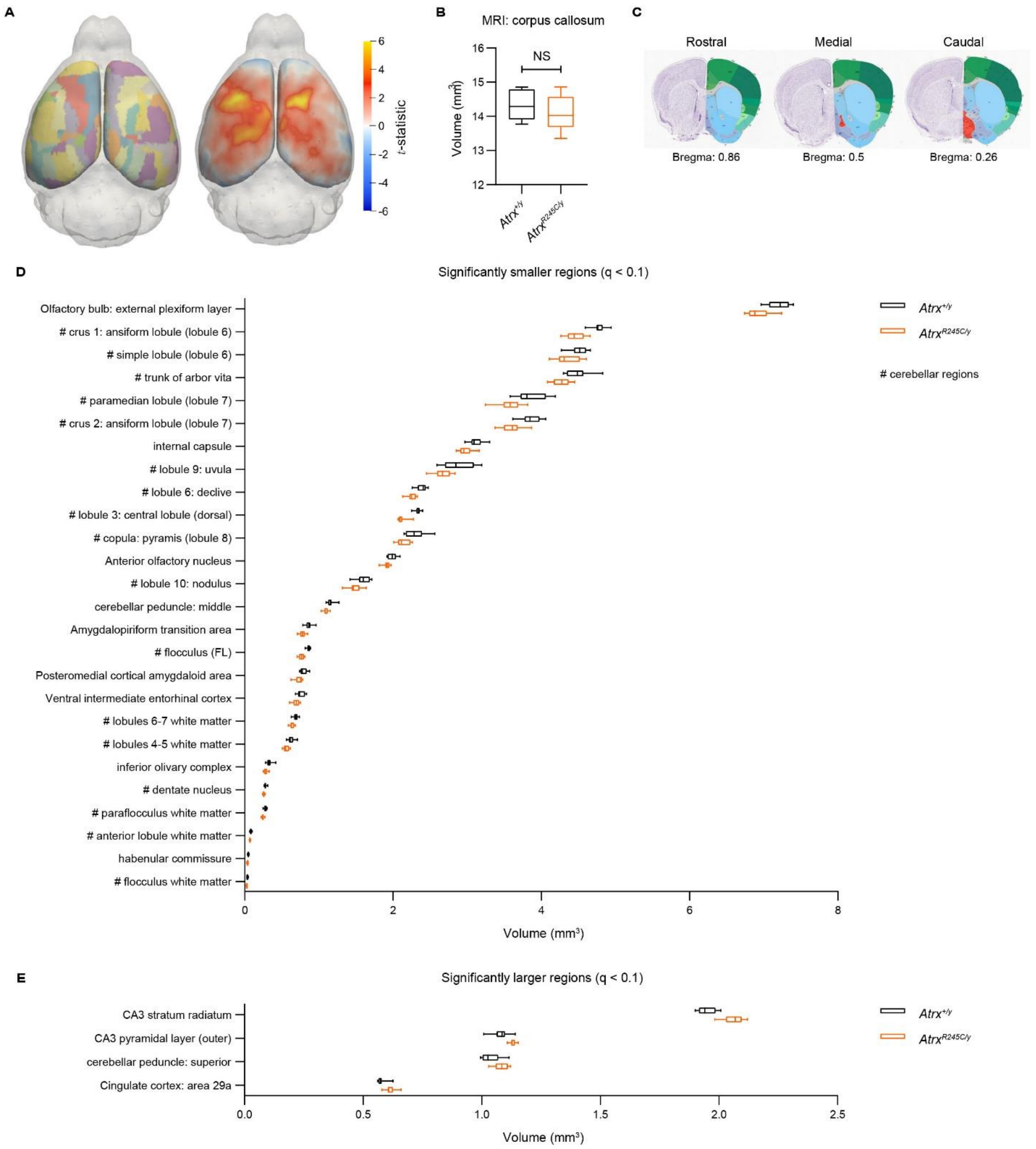

Figure S6. Changes in brain volume in *Atrx*$^{R245C/y}$ mice

A. Cortical thickness analysed by MRI at 9 weeks (WT n = 10; *R245C* n = 12). Top view showing a surface projection of the DSURQE atlas (left) and cortical thickness differences comparing R245C to WT, computed independently at each surface vertex and quantified by the t-statistic. **B.** Corpus callosum volume. Graph shows interquartile ranges and genotypes were compared using a t-test: P > 0.05. **C.** Diagram showing rostral, redial and caudal sections used to measure corpus callosum

thickness. **D-E.** Volumes of the significantly (q < 0.1) smaller (**D**) and larger (**E**) brain regions. Graphs show interquartile ranges with min-max whiskers.

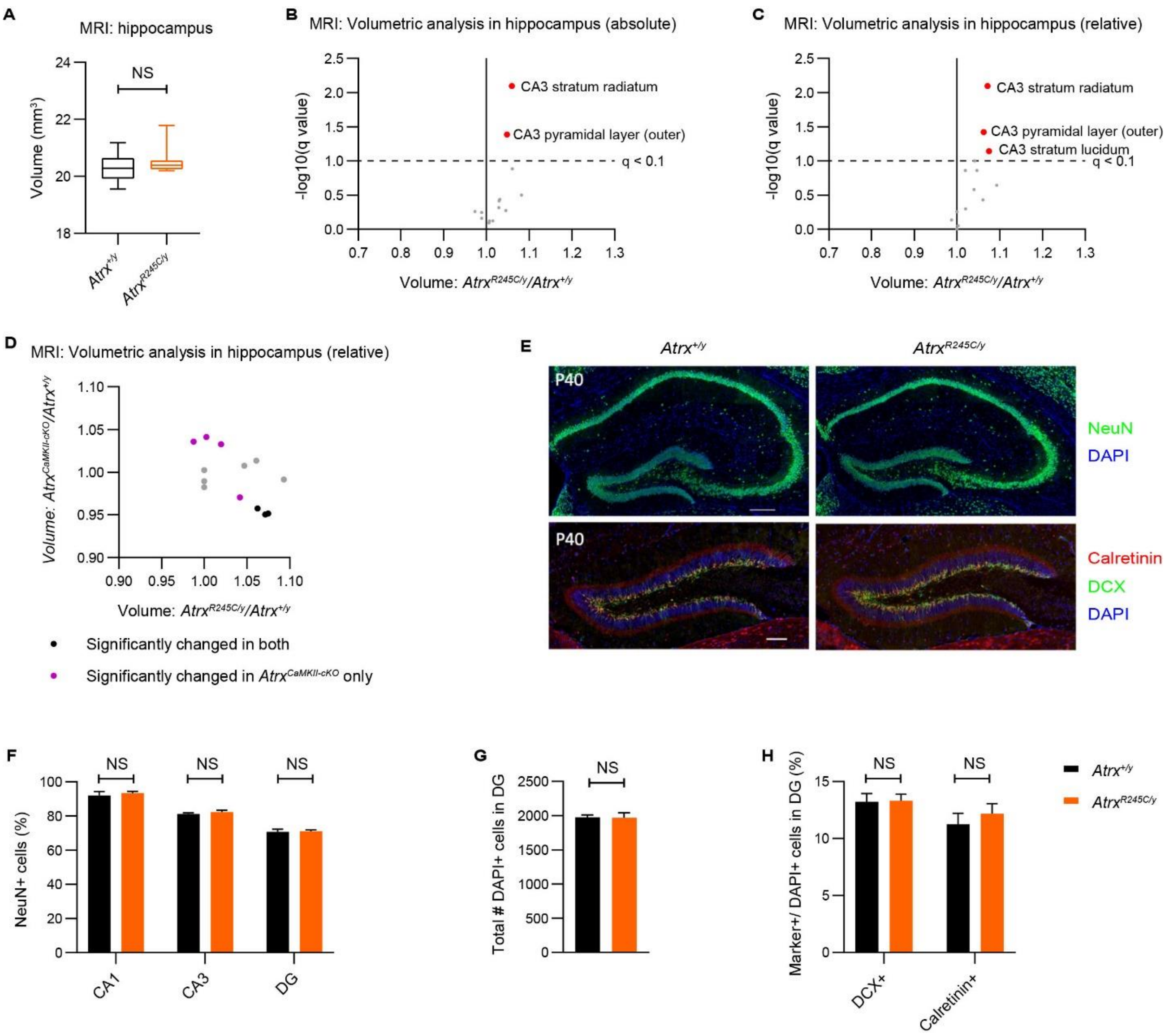

Figure S7. Hippocampal development is unaffected in *Atrx*$^{R245C/y}$ mice

A. Total hippocampal volume analysed by MRI at 9 weeks (WT n = 10; *R245C* n = 12). Graph shows interquartile ranges and genotypes were compared using a KS test: P > 0.05. **B-C.** Volcano plots of absolute (**B**; as in Figure 6C and F) and relative (**C**; as performed for *Atrx CaMKII-cKO* mice (ref. 10)) volumetric analysis of the 15 subregions of the hippocampus. Significantly increased (q < 0.1) regions are highlighted in red. **D.** Comparison of relative volumetric analysis in the hippocampus between *Atrx*$^{R245C/y}$ and *Atrx CaMKII-cKO* mice. **E.** Representative images of hippocampal (upper) and dentate gyrus (lower) sections from WT and *R245C* mice at P40. Hippocampal sections were stained with NeuN, a marker of mature neurons (green). Scale bar: 200 µm. Dentate gyrus sections were stained with calretinin (red), DCX (green) and DAPI. Scale bar: 100 µm. **F.** Percentage of NeuN+ cells for CA1, CA3 and dentate gyrus (DG) regions of the hippocampus. **G-H.** Total number

of DAPI+ cells (**G**) and percentage of calretinin+ and DCX+ cells (**H**) in the DG. **E-H.** N = 4 biological replicates per genotype. All graphs show mean ± S.E.M. and genotypes were compared using t-tests: all NS $P > 0.05$.
